# *Pteropus vampyrus* TRIM40 Is an Interferon-Stimulated Gene That Antagonizes RIG-I-like Receptors

**DOI:** 10.3390/v15112147

**Published:** 2023-10-25

**Authors:** Sarah van Tol, Adam Hage, Ricardo Rajsbaum, Alexander N. Freiberg

**Affiliations:** 1 Department of Microbiology and Immunology, University of Texas Medical Branch, Galveston, TX 77555, USA; sarah.vantol@nih.gov (S.v.T.); adam.hage@nih.gov (A.H.); 2 Center for Virus-Host-Innate-Immunity and Department of Medicine, RBHS Institute for Infectious and Inflammatory Diseases, New Jersey Medical School, Rutgers—The State University of New Jersey, Newark, NJ 07103, USA; 3 Institute for Human Infections and Immunity, Sealy & Smith Foundation, University of Texas Medical Branch, Galveston, TX 77555, USA; 4 Department of Pathology, University of Texas Medical Branch, Galveston, TX 77555, USA; 5 Center for Biodefense and Emerging Infectious Diseases, University of Texas Medical Branch, Galveston, TX 77555, USA

**Keywords:** tripartite motif (TRIM) proteins, RIG-I-like receptors, MDA5, RIG-I, Nipah virus (NiV), TRIM40, inflammatory responses in bats and humans, innate immunity, type-I interferons (IFNs)

## Abstract

Nipah virus (NiV; genus: Henipavirus; family: *Paramyxoviridae*) naturally infects Old World fruit bats (family *Pteropodidae*) without causing overt disease. Conversely, NiV infection in humans and other mammals can be lethal. Comparing bat antiviral responses with those of humans may illuminate the mechanisms that facilitate bats’ tolerance. Tripartite motif proteins (TRIMs), a large family of E3-ubiquitin ligases, fine-tune innate antiviral immune responses, and two human TRIMs interact with Henipavirus proteins. We hypothesize that NiV infection induces the expression of an immunosuppressive TRIM in bat, but not human cells, to promote tolerance. Here, we show that TRIM40 is an interferon-stimulated gene (ISG) in pteropodid but not human cells. Knockdown of bat TRIM40 increases gene expression of IFNβ, ISGs, and pro-inflammatory cytokines following poly(I:C) transfection. In *Pteropus vampyrus*, but not human cells, NiV induces TRIM40 expression within 16 h after infection, and knockdown of TRIM40 correlates with reduced NiV titers as compared to control cells. Bats may have evolved to express TRIM40 in response to viral infections to control immunopathogenesis.

## 1. Introduction

Several bat species serve as the natural reservoir host for multiple virulent viruses that have been shown to spillover into humans [1]. Henipavirus (HNV; family: *Paramyxoviridae*), including Nipah virus (NiV) and Hendra virus (HeV), infection in bats does not cause overt clinical disease [2,3,4]. Conversely, HeV or NiV infection in humans and other mammals can be lethal with case fatality rates reaching 50% or higher [5,6]. While the majority of HNV studies focus on the pathogenesis in amplification and spillover hosts [7,8,9], countermeasure development [10,11,12], and virus ecology within *Pteropus* populations [3,13,14,15,16,17,18,19,20,21], there are large gaps in our understanding of the within-host factors that influence the success of *Pteropus* bats as an existing reservoir and need to be addressed. Comparing bat antiviral responses and bat–viral protein interactions with those of humans may illuminate the mechanisms that facilitate bats’ tolerance.

Currently, multiple hypotheses have been proposed to explain bats’ tolerance to viral infection but with limited experimental evidence. One proposed explanation of bats’ tolerance to viruses is the constitutive expression of interferon-alpha (IFNα) in some bat species [22]. The IFN-stimulated genes (ISGs) basally expressed, due to constant IFNα expression, are predicted to be involved in antiviral and DNA repair functions but not in pro-inflammatory responses [22]. Reduced pro-inflammatory signaling in bats after virus recognition may limit the immunopathogenic side effects observed in other mammalian species [23,24,25]. Other hypotheses include the co-evolution of bats and viruses to allow viral persistence and shedding without causing death or overt disease, and the evolution of powered flight in bats selected for an immune system with unique immunosuppressive mechanisms that impair virus clearance as a trade-off [26,27,28,29,30]. Many factors likely contribute to bats’ tolerance to virus infections, and these hypotheses are not mutually exclusive. The molecular mechanisms underlying tolerance and the significance of those factors in the context of bat-borne virus infection are unknown.

We are interested in elucidating differences between bats and humans in their ability to regulate the antiviral and pro-inflammatory branches of the type-I interferon (IFN-I) pathways [31]. HeV and NiV encode proteins that potently antagonize human IFN-I production and signaling, but there is conflicting evidence regarding the henipaviral inhibition of bat IFN-I pathways [32,33]. Clarifying pteropine IFN-I–henipaviral interactions and elucidating the differences in IFN-I regulation between pteropid bats and humans could prove crucial in the development of strategies to treat patients infected with HNVs. Modelling HNV infection in pteropodid bat cells may allow the identification of factors contributing to tolerance.

Several tripartite motif proteins (TRIMs), a large family of E3-ubiquitin ligases, regulate antiviral immune responses [34,35,36]. TRIMs fine-tune the innate antiviral immune response with some TRIMs promoting an antiviral, pro-inflammatory response while other TRIMs repressing inflammatory pathways to prevent immunopathogenesis [34]. Additionally, NiV encodes proteins that antagonize two human TRIMs [37,38]. Previously, we reported that human TRIM6, a positive regulator of IFN-I pathways [39], is targeted for degradation by NiV matrix protein (NiV-M) [37], and another group demonstrated NiV accessory protein V antagonizes TRIM25-mediated RIG-I activation [38].

Here, we investigated the potential for TRIM40 to differentially regulate NiV infection in human and bat cells. We re-analyzed previous next-generation RNA sequencing studies [40,41,42] and identified TRIM40 to be expressed in pteropodid cells in response to IFN-I stimulation. In contrast, while TRIM40 has not been identified as an ISG in other species [40] including mice and humans [43], human and murine TRIM40 antagonizes the RNA recognition receptors MDA5 and RIG-I, resulting in impaired IFN-I induction and pro-inflammatory cytokine production [44,45]. We hypothesized that pteropid TRIM40 is an anti-inflammatory ISG and found that bat TRIM40 is IFN-responsive in two distantly related species, antagonizes RLR function, and is induced early after NiV infection. Despite differential regulation of expression, both human and bat TRIM40s can promote NiV replication. Future studies are needed to investigate whether bat’s TRIM40 can contribute to viral tolerance in vivo.

## 2. Materials and Methods

### 2.1. Cell Lines

Vero (ATCC CCL-81; African green monkey, *Chlorocebus aethiops*), A549 (ATCC CCL-185; human lung carcinoma epithelial cells), 293T (ATCC CRL-3216; human fetal kidney), PVK4 [32] (Malayan flying fox, *Pteropus vampyrus*, epithelial kidney cell line immortalized with telomerase), and ZFBK13-75 [46] (Straw-colored fruit bat, *Eidolon helvum*, epithelial kidney cell line transformed with SV40 L) cells were maintained in 1× DMEM with 10% FBS and incubated at 37 °C, 5% CO_2_. Vero CCL81 cells were used for Nipah virus (NiV) plaque assay. 293T and ZFBK13-75 cells were used for transfection experiments. A549, PVK4, and ZFBK13-75 cells were used for immune stimulation and NiV infection experiments.

To generate TRIM40 CRISPR knockout PVK4 cells, lentiCRISPRv2 puro (Plasmid #98290-Addgene) plasmid was cut with BsmBI, and TRIM40-targeting or control sgRNA oligo pairs (Table 1) were ligated into the plasmid. The lentiCRISPRv2 puro plasmids were transfected with Lipofectamine 3000 (Invitrogen, Waltham, MA, USA) according to manufacturer’s instructions. Two days after transfection, cells were replated in DMEM with puromycin for one week before serial dilution and then plated in 96-well plates to isolate clonal populations.

### 2.2. Plasmids

The FLAG-tagged human MDA5, RIG-I, and IRF3 and His-ubiquitin constructs are present in the pCAGGS backbone [39]. The human HA-TRIM40 in pcDNA3.1 plasmid was subcloned into pCAGGS-HA using primers listed in Table 1. First-strand cDNA from PVK4 cells stimulated with U-IFN (PBL Bioscience, Piscataway, NJ, USA) or transfected with 1 μg/mL LMW poly(I:C) was generated using the RevertAid First Strand cDNA Synthesis kit (Thermo Fisher, Waltham, MA, USA), and it was used as the template to clone open reading frame sequences of *Pteropus vampyrus* RIG-I, MDA5, and TRIM40 (primers listed in Table 1). The TRIM40 catalytic mutant, C14.17A, was generated using primers (Table 1) containing point mutations and cloned into the pCAGGS-HA vector. The PCR reactions were conducted using the AccuPrime Taq DNA Polymerase, high fidelity kit (Invitrogen). All plasmid sequences were confirmed using Sanger sequencing (UTMB Molecular Genomics, Galveston, TX, USA).

### 2.3. Viruses

Recombinant Nipah virus (199901924 Malaysia prototype strain) expressing enhanced GFP in the nucleoprotein and phosphoprotein intergenic region (rNiV-GFP) [47] was used for all infection experiments. All manipulations with infectious NiV were performed in the Robert E. Shope and Galveston National Laboratory Biological Safely Level 4 facilities at UTMB.

### 2.4. Virus Infections and Plaque Assays

Cells were plated in 10% FBS DMEM 16 h prior to infection. The virus inoculum was prepared in 2% FBS DMEM. A portion of the inoculate was saved for back titration. At the time of infection, the medium was removed, and 100 μL of the inoculum was added. The cells were incubated with the inoculum for 1 h at 37 °C, 5% CO_2_, and rocked every 15 min. The cells were washed three times with 1× DPBS (Corning) to remove the inoculum, and fresh 2% FBS DMEM was added. At the indicated time points, supernatants and RNA were collected for titration and qPCR, respectively. An Olympus (IX73) microscope was used to take fluorescence and bright field images. Viral titers were determined using a plaque assay on Vero (CCL-81) cells, as previously described [37].

### 2.5. Transfections and Immunoprecipitations

293T or ZFBK13-75 cells were plated in 6-well plates (400,000 cells/well) in 10% FBS DMEM for 16 h, followed by transfection using TransIT-LT1 (Mirus, Madison, WI, USA) for 293T or Lipofectamine 3000 for ZFBK13-75, following the manufacturer’s recommendations. After 26–30 h, transfected 293T or ZFBK13-75 cells were lysed in RIPA buffer with complete protease inhibitor (Roche, Basel, Switzerland), n-ethylmaleimide (NEM), and iodoacetamide (IA) (RIPA complete). Lysates were cleared at 25,200× *g* for 20 min at 4 °C, and 10% of the clarified lysate was added to 2× Laemmli sample buffer (BioRad, Hercules, CA, USA) with 5% beta-mercaptoethanol and boiled at 95 °C for 10 min to generate whole cell extracts (WCE). The remaining clarified lysate was mixed with 7.5 μL of anti-HA-Agarose beads (Sigma, Burlington, MA, USA) or anti-FLAG-Agarose beads (Sigma) and incubated at 4 °C overnight on a rotating platform. The beads were washed seven times with RIPA buffer with IA and NEM before boiling in 2× Laemmli buffer (HA and FLAG co-IP).

### 2.6. Protein Purification

To collect purified HA-TRIM40, we transfected 293T cells and immunoprecipitated them with anti-HA beads as described above, prior to peptide elution. After the seven washes in 1× TBS-T, beads were washed once in peptide elution buffer (10 mM TRIS pH 7.4 and 150 mM NaCl in nuclease free water (NF H_2_O)) without peptide. The protein was then eluted in 15 μL of peptide elution buffer with HA (1 mg/mL) peptide three times. The peptide purified protein was aliquoted and stored at −80 °C until use.

### 2.7. Immune Stimulations

For immune stimulations, A549, PVK4, and ZFBK13-75 cells were plated in 12-well plates (200,000 cells/well) in 10% FBS DMEM for 16 h. Low-molecular weight poly(I:C) (Invitrogen) was diluted in OptiMEM to 10 μg/mL, and Lipofectamine 2000 (Invitrogen) was added (0.5 μL Lipofectamine 2000:1 μg LMW poly(I:C)) and incubated at room temperature for 10 min. A 100 μL volume of LMW poly(I:C) was added to each well (final concentration 1 μg/mL). Universal type I IFN (U-IFN) was diluted in OptiMEM to 5000 U/mL, and 100 μL was added to each well (final concentration: 500 U/mL).

### 2.8. Western Blots

Protein samples were run on 4–15% or 7.5% Mini-PROTEAN- or Criterion-TGX Precast Gels (Bio-Rad, Hercules, CA, USA). The proteins were then transferred onto methanol-activated Immun-Blot PVDF membrane (Bio-Rad), and the membrane was blocked with 5% Carnation powdered skim milk (Nestle, Vevey, Vaud, Switzerland) in 1× TBS-T (blocking buffer) for 1 h. Primary antibodies were prepared in 2% bovine serum albumin 1× TBS-T with 0.02% sodium azide to the appropriate dilution: anti-FLAG (Sigma) 1:2000, anti-HA (Sigma) 1:2000, anti-His (Sigma) 1:2000, phosphorylated TBK1 S172 (Epitomics, Burlingame, CA, USA) 1:1000, total TBK1 (Novus Biologicals, Centennial, CO, USA) 1:1000, phosphorylated STAT1 Y701 (Cell Signaling, Danvers, MA, USA) 1:1000, total STAT1 (Cell Signaling) 1:1000, phosphorylated STAT2 Y690 (Cell Signaling) 1:1000, total STAT2 (Cell Signaling) 1:1000, anti-ubiquitin (Enzo, Farmingdale, NY, USA) 1:1000, anti-tubulin (Sigma) 1:2000, and anti-actin (Abcam, Cambridge, UK) 1:2000. The next day, the blot was washed in 1× TBS-T prior to incubation with HRP-conjugated goat-anti-rabbit (GE Healthcare, Chicago, IL, USA) or goat-anti-mouse (GE health care) for 1 h. The blot was then washed and developed using Pierce ECL Western Blotting Substrate (Thermo Fisher) or SuperSignal West Femto Maximum Sensitivity Substrate (Thermo Fisher).

### 2.9. RNA Extractions and Quantitative PCR

Cells were lysed in Trizol (Thermo Fisher) or Tri-reagent (Zymo Research, Irvine, CA, USA) and processed using the Direct-zol RNA kit (Zymo Research). For the standard qPCR reactions, cDNA was synthesized using the High-Capacity cDNA Reverse Transcription Kit (Applied Biosystems, Waltham, MA, USA), following the manufacturer’s instructions. The qPCR master mixes were prepared with *iTaq* Universal SYBR Green (Bio-Rad). The qPCR reactions were carried out using a CFX384 instrument (Bio-Rad). The relative mRNA expression levels were analyzed using CFX Manager software (Bio-Rad version 3.1). The change in the threshold cycle (ΔCT) was calculated, with the GAPDH gene serving as the reference mRNA for normalization. Where indicated, fold induction was calculated using ΔΔC_T_ of stimulated/ΔΔC_T_ mock transfected. The primers used to assess gene expression are listed in Table 2.

### 2.10. Knockdown Experiments

Transient knockdown of endogenous TRIM40 in A549 or PVK4 cells was performed in 12-well plates. For A549 cells, 20 pmol of Smartpool ON-TARGETplus nontargeting (catalog number D-001810-10-05) or ON-TARGETplus TRIM40 (catalog number LQ-007129-00-0005) siRNA (Dharmacon, Lafayette, CO, USA) was transfected with Lipofectamine RNAiMAX (Invitrogen, Lafayette, CO, USA) according to the manufacturer’s instructions. For PVK4 cells, 20 pmpl of TRIM40 targeting siRNA (G.G.A.A.G.G.A.G.G.C.C.C.U.A.U.U.A.G.A.U.U) or a scrambled, non-targeting control (G.G.G.C.U.G.G.A.G.U.C.A.A.A.U.A.C.G.A) was transfected with RNAiMAX. Cells were transfected with siRNA 24 h prior to infection. TRIM40 knockdown efficiency was evaluated with qRT-PCR.

### 2.11. Quantification and Statistical Analysis

All analyses were performed in GraphPad Prism (Version 7.04). Heat maps were also generated with GraphPad Prism. Densitometry of Western blots was performed using ImageJ software (1.53t). Statistical tests, measures of statistical significance, and replication information are specified in the respective figure legends. One-way ANOVA with Tukey’s post-test was used for comparing three or more groups and a student’s *t*-test for comparing two groups. Specific statistical tests and significance cut-offs are reported in the figure legends where appropriate.

## 3. Results

### 3.1. Pteropodid TRIM40 Is an IFN-Stimulated Gene (ISG)

**Figure 1:**
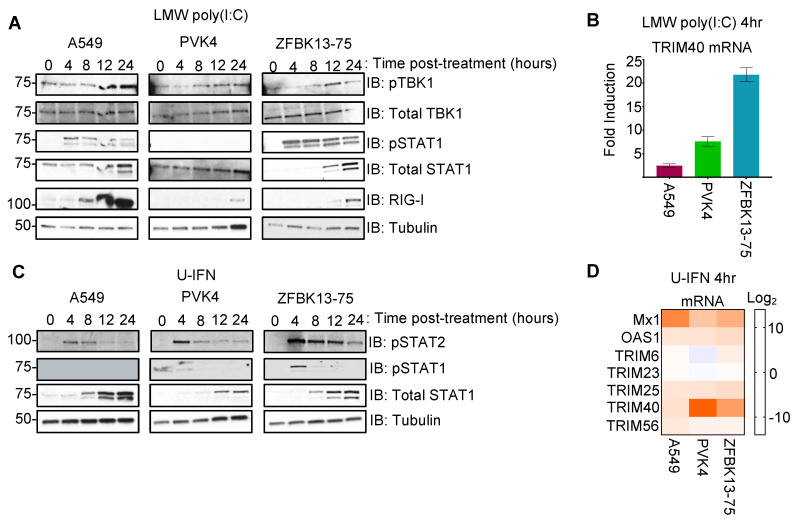
Pteropodid TRIM40 is an interferon-stimulated gene. (**A**,**B**) Transfection of A549 (human lung), PVK4 (*Pteropus vamyprus* kidney), and ZFBK13-75 (*Eidolon helvum* kidney) cells with 1 μg/mL of low-molecular weight (LMW) poly(I:C). (**A**) Immunoblots with antibodies specific to interferon induction regulators (pTBK1 and pSTAT1) and interferon-stimulated genes (total STAT1 and RIG-I). (**B**) At four hours post-stimulation, RNA was collected for quantitative PCR of the TRIM40 gene. Fold induction of poly(I:C)-stimulated samples relative to mock-transfected samples is shown. (**C**,**D**) A549, PVK4, and ZFBK13-75 cells were stimulated with 500 U/mL of universal type I interferon (U-IFN). (**C**) Cell lysates were collected for Western blot to probe for activated STAT1 (pSTAT1 Y701) and STAT2 (pSTAT2 Y690) as well as their total protein levels. (**D**) At four hours post-stimulation, RNA was collected for qPCR, and log_2_ fold change is presented for canonical ISGs (Mx1, OAS1, TRIM25, and TRIM56), non-IFN-regulated TRIM23, and the gene of interest, TRIM40.

We first tested whether pteropodid TRIM40 is interferon inducible and how it compares with the expression of human TRIM40. Human cells (A549-lung carcinoma) and bat cells from *Pteropus vampyrus* (PVK4- kidney fibroblast) or *Eidolon helvum* (ZFBK13-75A- kidney fibroblast) were transfected with low-molecular weight poly(I:C), a double-stranded RNA mimic, to stimulate IFN induction. We observed the activation of different signaling factors involved in the IFN-I induction and signaling pathways, including the phosphorylation of TBK1 (pTBK1 S172) and pSTAT1 Y701, thereby increasing levels of total STAT1 and RIG-I (both are ISGs), in each of the cell lines (Figure 1A). Soon after the poly(I:C) treatment, TRIM40 gene expression was induced in both fruit bat cells but not in the human cells (Figure 1B).

To assess more directly whether TRIM40 is an ISG, the bat and human cell lines were stimulated with universal type I IFN (U-IFN). As expected, we found an increase in STAT1 phosphorylation early after the IFN treatment, which was followed by an increased expression of total STAT1 in both human and bat cells (Figure 1C), indicating that these cell lines are responsive to IFN-I treatment. Transcription of the canonical ISGs, Mx1, OAS1, and TRIM25 was induced, whereas TRIM23, which is not known to be an ISG, was unchanged following U-IFN treatment in each of the cell lines (Figure 1D). Although all cells tested are IFN-I responsive, we observed a strong induction of TRIM40 gene expression in both bat cells but not in the human cells (Figure 1D). These observations support that TRIM40 is an ISG in pteropodid bat but not human cells.

### 3.2. Pteropus vampyrus TRIM40 Antagonizes Type I Interferon and Pro-Inflammatory Cytokine Gene Expression

**Figure 2:**
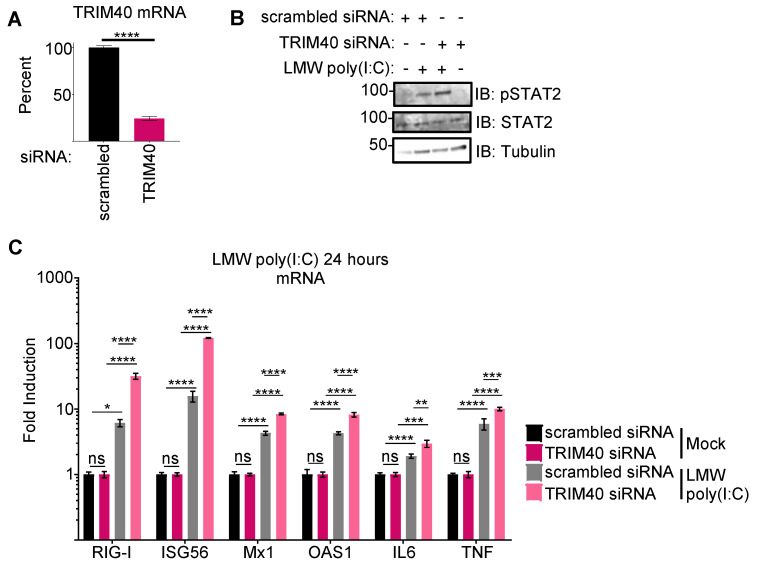
Pteropus vampyrus TRIM40 antagonizes type I interferon and pro-inflammatory cytokine gene expression. siRNA targeting *Pteropus vampyrus* TRIM40 or scrambled control was transfected in PVK4 cells 48 h prior to the transfection of 1 μg/mL of low-molecular weight (LMW) poly(I:C). (**A**) The RNA collected at 24 h post-poly(I:C) transfection shows knockdown efficiency. (**B**) Lysates collected in laemmli were used for Western blot to measure activated (pSTAT2) and total STAT2. (**C**) RNA from lysates was used to measure fold-induction of ISGs (RIG-I, ISG56, Mx1, and OAS1) and pro-inflammatory cytokines (IL6 and TNFα). *p* values are reported as * < 0.05, ** < 0.01, *** < 0.001, **** < 0.0001.

Next, we tested whether bat TRIM40 functions analogously to human TRIM40 as a negative regulator of RIG-I-like receptors [44,45]. We designed siRNA targeting TRIM40 or generated a scrambled sequence. The knockdown efficiency was over 75% at the RNA level (Figure 2A). Unfortunately, commercially available TRIM40 antibodies do not cross-react with *Pteropus* TRIM40, and we were not able to assess knockdown at the protein level. Following TRIM40 knockdown in PVK4 cells, we observed stronger STAT2 phosphorylation (Figure 2B) and higher levels of both ISGs (RIG-I, ISG56, Mx1, and OAS1) and pro-inflammatory cytokines (IL6 and TNFα), as compared to the scrambled siRNA controls (Figure 2C). No effects were observed in knockdown cells in non-stimulated (mock) conditions. These data support that *P. vampyrus* TRIM40 functions as a negative regulator of pattern recognition receptor (PRR) pathways similar to human TRIM40, upon dsRNA stimulation.

### 3.3. Pteropus vampyrus TRIM40 Interacts with and Ubiquitinates RIG-I-like Receptors RIG-I and MDA5

**Figure 3:**
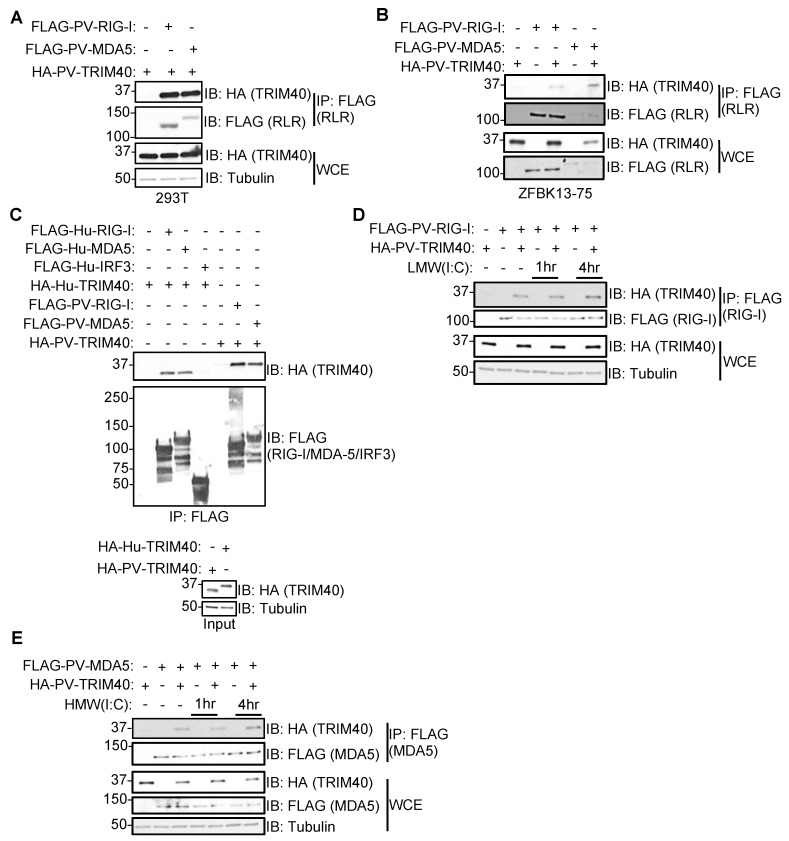
Pteropus vampyrus TRIM40 interacts with RIG-I-like receptors RIG-I and MDA5. Cells were co-transfected with *Pteropus vampyrus* HA-PV-TRIM40 and FLAG-PV-RIG-I-like receptor (RLR) RIG-I or MDA5 for 30 h in 293T (**A**) or ZFBK13-75 cells (**B**). The whole cell extracts (WCE) were used to check levels of expression and immunoprecipitated (IP) with anti-FLAG antibody beads. (**C**) 293T cells were transfected with human (Hu) or PV HA-TRIM40 and partially purified using HA peptide. Separate 293T cells were transfected with Hu- or PV-RLRs or HA-Hu-IRF3. Lysates from FLAG-protein expressing cells were immunoprecipitated with FLAG beads and washed prior to the addition of HA-purified TRIM40. (**D**) ZFBK13-75 cells were co-transfected with FLAG-PV-RIG-I and HA-PV-TRIM40 for 24 h and then stimulated with 1 μg/mL low-molecular weight (LMW) poly(I:C) for 1 or 4 h, and lysates were used for WCE or IP: FLAG. (**E**) ZFBK13-75 cells were co-transfected with FLAG-PV-MDA5 and HA-PV-TRIM40 for 24 h and then stimulated with 1 ug/mL high-molecular weight (HMW) poly(I:C) for 1 or 4 h, and lysates were used for WCE or IP: FLAG.

Next, we asked whether *Pteropus* TRIM40 can interact with the RLRs RIG-I and MDA5. We co-transfected FLAG-RLRs with HA-TRIM40 in human 293T (Figure 3A) or bat cells (Figure 3B) and found that *P. vampyrus* TRIM40 co-immunoprecipitated with both RIG-I and MDA5 in both cell types.

To test the interaction between bat TRIM40 and RLRs more directly, we performed a cell-free interaction assay by mixing cell lysates. Cells were co-transfected with either HA-TRIM40 from bat or human source, and FLAG-RLR or IRF3 was used as a negative control. Both human and *Pteropus* TRIM40s interacted with their homologous RLRs, whereas human IRF3 did not interact with human TRIM40 and served as a negative control (Figure 3C). Furthermore, HA-TRIM40′s interaction with FLAG-RIG-I and -MDA5 was also observed following stimulation with low- (LMW) or high-molecular weight (HMW) poly(I:C), respectively (Figure 3D,E).

**Figure 4:**
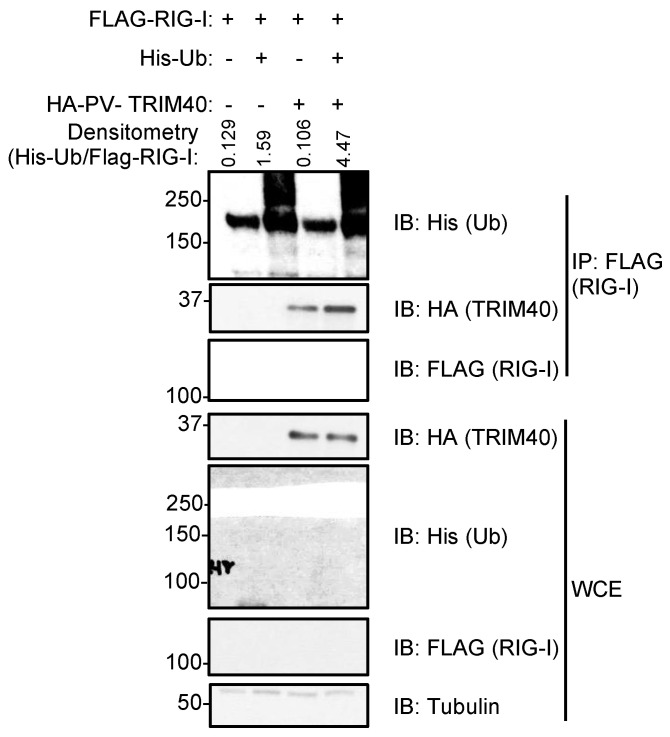
*Pteropus vampyrus* TRIM40 ubiquitinates RIG-I-like receptors RIG-I and MDA5.

We then tested whether *Pteropus* TRIM40 is a functional E3-ubiquitin ligase and can mediate the ubiquitination of RIG-I. We found that the co-immunoprecipitation of His-ubiquitin (Ub) with FLAG-RIG-I is enhanced in the presence of overexpressed HA-PV-TRIM40 (Figure 4, IP).

ZFBK13-75 cells were transfected with *Pteropus vampyrus* (PV) HA-TRIM40, His-ubiquitin (Ub), and a FLAG-RIG-I. Whole cell extracts (WCE) were added to laemmli to measure their expression or immunoprecipitated levels with anti-FLAG antibody-coated beads. The densitometry was performed using ImageJ software, and the relative values for His-Ub in the IP were normalized by the relative values of FLAG-RIG-I in the IP.

### 3.4. Nipah Virus Infection Induces TRIM40 Expression in Pteropus vampyrus Cells

**Figure 5:**
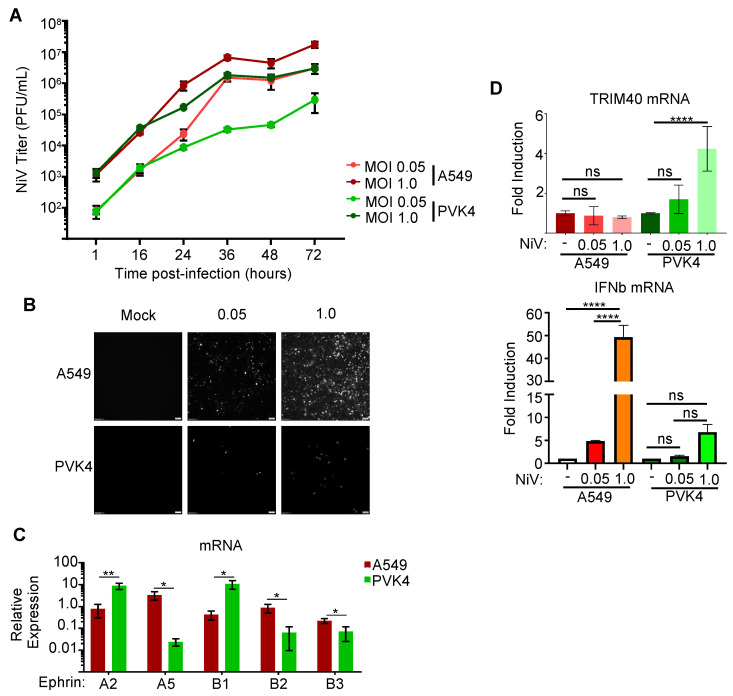
Nipah virus infection induces TRIM40 expression in *Pteropus vampyrus* cells. (**A**,**B**) A549 and PVK4 cells were infected with a recombinant Nipah virus (NiV) that express GFP at a multiplicity of infection of 0.05 or 1.0, and supernatants were collected throughout the 72 h post-infection period (**A**) with fluorescence microscopy images taken at 36 h (**B**). (**C**) Ephrin A and B gene expression in unstimulated PVK4 and A549 cells reported as relative expression normalized to GAPDH. (**D**) TRIM40 gene expression in A549 and PVK4 cells 16 h post-infection (h.p.i) with MOI 0.05 or 1.0 rNiV-GFP reported as fold induction (top panel), and IFNβ mRNA expression (bottom panel). *p* values are reported as * < 0.05, ** < 0.01, **** < 0.0001.

We next analyzed the role of TRIM40 in the context of infection. *Pteropus* or human (A549) cells were infected with a recombinant NiV-Malaysia expressing GFP (rNiV-GFP) [47] with either a low (0.05) or high (1.0) multiplicity of infection (MOI) to identify comparable infection conditions between the two cell lines. The PVK4 cells infected at MOI 1.0 and human cells infected at MOI 0.05 exhibited similar production of the infectious virus (Figure 5A) despite a lower GFP expression in PVK4 cells (Figure 5B). The difference in viral dose needed to produce similar levels of NiV production between the cell lines may be partially attributed to the relatively lower levels of the NiV receptors ephrin-B2 and -B3 in the PVK4 cells (Figure 5C). We then measured TRIM40 gene expression in these cells and observed a dose-dependent induction of TRIM40 expression in PVK4, but not A549, cells within 16 h of infection (Figure 5D, top panel). In contrast, IFNb mRNA was significantly induced upon NiV infection in A549 cells but only marginally in bat cells, suggesting that bat TRIM40, and not human TRIM40, is a highly sensitive ISG during NiV infection.

### 3.5. TRIM40 Promotes Virus Replication in Bat and Human Cells

**Figure 6:**
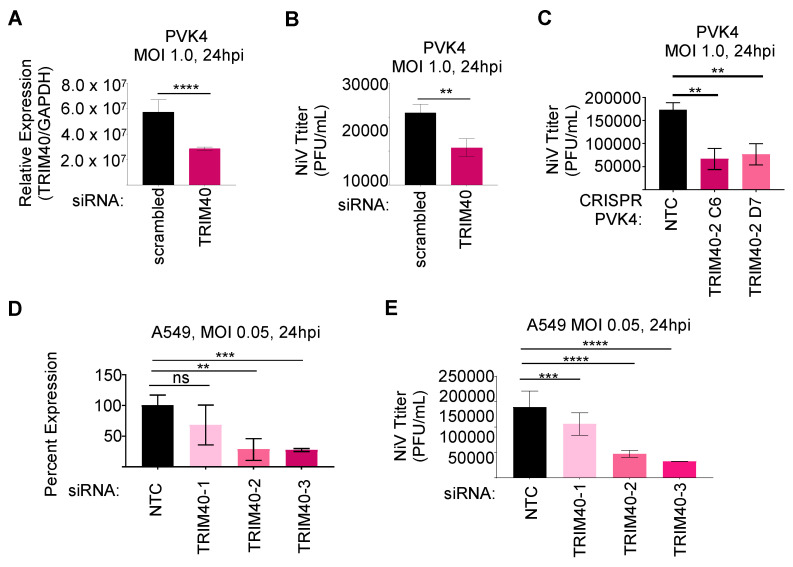
TRIM40 is proviral in bat and human cells. PVK4 cells infected with recombinant Nipah virus (rNiV) expressing GFP at a multiplicity of infection of 1.0 and measured knockdown efficiency of TRIM40 (**A**) and NiV titer (**B**) at 24 h post-infection. CRISPR-knockout or control PVK4 cells were infected with rNiV-GFP MOI 1.0 for 24 h to measure titer (**C**). A549 cells infected with rNiV-GFP at MOI 0.05 and measured knockdown efficiency of TRIM40 (qPCR) (**D**) and NiV titer (**E**) at 24 h post-infection. *p* values are reported as ** < 0.01, *** < 0.001, **** < 0.0001.

We then tested the effects of TRIM40 depletion on NiV replication in human and *Pteropus* cells. Using siRNA to knockdown TRIM40 in PsVK4 cells (Figure 6A), although the knockdown was only partially efficient, we observed reduced NiV titers as compared to control cells, at 24 h.p.i. (Figure 6B). The same attenuation of NiV occurred in TRIM40 knockout PVK4 cells (Figure 6C). Knockdown of human TRIM40 in A549 cells using siRNA (Figure 6D) also resulted in reduced NiV replication (Figure 6E). These results suggest that TRIM40 could be induced in *Pteropus* cells to prevent the overactivation of the innate antiviral response, but the proviral role of TRIM40 is conserved in both human and bat cells.

## 4. Discussion

Pteropodid bats are the reservoir host of several henipaviruses, and the majority of data supports that these Old World fruit bats host these viruses without experiencing overt clinical disease [2,3,4,13], while infection in other susceptible mammalian hosts tends to cause severe or fatal disease [5,48,49]. Identifying the within-host factors that modulate differential disease outcomes between reservoir and non-reservoir hosts is important and could lead to the development of therapeutic strategies.

Multiple hypotheses have been proposed to explain bats’ ability to support diverse viruses without causing apparent disease. Many theories spur from the expectation that bats’ evolution of powered flight applied strong selective pressures in pathways intersect with immunity [28]. The two main, non-mutually exclusive groups of hypotheses are (1) bats evolved unique restrictive factors limiting viral burden during infection, and (2) bats evolved tolerogenic mechanisms preventing host-inflicted damage during infection. Considering the immense diversity of the bat order Chiroptera (21 families, 232 genera, and 1452 species [50]), and the viruses they host [51], unique within-host factors likely contribute to any given species’ capacity to serve as a reservoir for a given virus. Here, we investigated the potential tolerogenic role of pteropid TRIM40 in regulating Nipah virus infection.

Human and mouse TRIM40s negatively regulates the innate antiviral response [44,45]. We hypothesized that pteropid TRIM40 functions analogously, facilitating anti-inflammatory and regulatory functions and could contribute to cellular tolerance to NiV infection. We first confirmed that TRIM40 expression is IFN-I inducible in two divergent Old World fruit bats’, *Pteropus vampyrus* (PVK4) and *Eidolon helvum* (ZFBK13-75), cell lines. As expected, human cells did not induce TRIM40 expression following U-IFN stimulation or poly(I:C) transfection. In mice, TRIM40 expression decreases during Sendai virus infection [44]. We also showed that following NiV infection, TRIM40 gene expression is induced in PVK4 but not in A549 cells. A comparison of ten species ISG repertoires only identified TRIM40 as an ISG in *P. vampyrus* despite the inclusion of a microbat, *Myotis lucifugus*, in the analysis [40]. Future studies are needed to determine in which bat families TRIM40 is an ISG.

**Figure 7:**
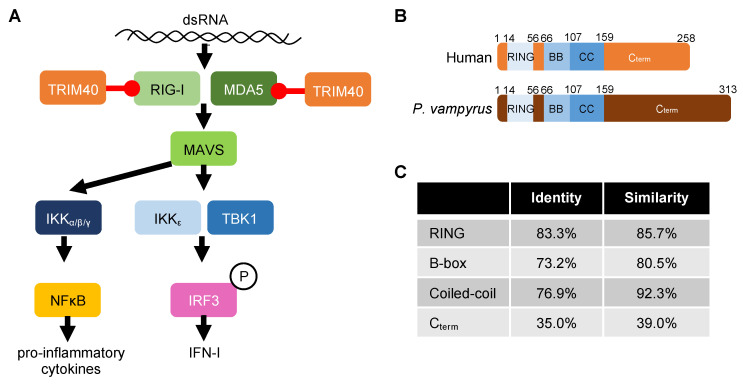
TRIM40 function and conservation. (**A**) Schematic of TRIM40′s role in regulating the RIG-I-like receptor pathway in pteropid bat and human cells. (**B**) Protein sub-domain organization of *Pteropus vampyrus* and human TRIM40. (**C**) Percent identity and similarity of *P. vampyrus* and human TRIM40.

Previous studies have shown that human TRIM40 facilitates lysine (K) 27- and K48-linked ubiquitin conjugation onto the CARD domains of RLRs promoting their proteosomal degradation [44,45]. Our experiments also demonstrate that bat TRIM40 acts as a negative regulator of the IFN-I induction pathway. This antagonism likely occurs through TRIM40-mediated ubiquitination and subsequent antagonism of RIG-I and MDA5 (Figure 7A), since we observe an interaction between *P. vampyrus* TRIM40 and the RLRs and an enhancement of RLR ubiquitination when co-expressed with TRIM40. Furthermore, the co-expression of the *P. vampyrus* TRIM40 catalytic mutant with the RLRs did not increase the amount of RLR-associated ubiquitin. Human TRIM40 (258 amino acids) and *P. vampyrus* TRIM40 (313 amino acids) share similar protein organization with conserved RING, B-box, coiled-coil (CC), and C-terminal sub-domains, but their sequence identity is only 56.2% (Figure 7B). The poor conservation is attributable, primarily, to differences in their C-terminal domains (35% identity). Importantly, the CC domain, which mediates human TRIM40′s interaction with MDA5 and RIG-I [44], is well-conserved (76.9% identity; 92.3% similar) (Figure 7C). Many TRIM family members use their C-terminal regions to interact with their ubiquitination targets and/or influence subcellular localization [52,53,54], and the CC domain typically regulates multimerization [55]. Based on this finding, we would expect species with conserved CC sequence and/or charge to retain TRIM40–RLR interaction.

Despite the species-specific differences in TRIM40 induction following NiV infection, we observed a proviral role of TRIM40 in both bat and human cells. Although these results suggest that pteropid TRIM40 does not mediate cellular tolerance, we cannot rule out that TRIM40 may contribute to tolerance at the organism level. We expected to identify whether TRIM40 promotes tolerance using the cell culture system based on viral load and cell death, but an in vivo system may be needed to draw conclusions on tolerogenic function. Alternatively, a different cell type may provide a clearer answer.

The continued study of within-host factors that mediate bats’ capacity to support viruses without causing overt disease is needed to advance our understanding of tolerance. The development of additional resources such as cell lines from multiple cell types, annotated genomes from additional species, and access to captive bats for in vivo studies will advance the field’s capacity to address these questions more effectively. Overall, we found that TRIM40-mediated antagonism of RLR-induced antiviral immunity is conserved in *Pteropus vampyrus*, type I IFN- and virus-induced TRIM40 expression is species-specific, and TRIM40 is proviral during Nipah virus infection.

Some limitations from our study include that we were only able to investigate the role of TRIM40 using kidney cell lines from *Pteropus vampyrus* and *Eidolon helvum* due to the limited availability of cell lines and lack of accessibility to live bats for the generation of additional cell lines. Ideally, the cell lines would be compared to human kidney cells, but preliminary studies of human kidney (293T and 769P) and lung (A549) cell lines found henipavirus replication, IFN-I induction, and signaling kinetics between A549 and the bat cells to be the most comparable. Ideally, we would have included *E. helvum* TRIM40 in the molecular components of the paper, but we were unable to successfully clone TRIM40 from *E. helvum* cells. The study only investigates TRIM40 as an ISG in two families of Old-World fruit bats, and it would be interesting to assess if the ISG status is conserved across Chiropteran families. The conclusions that we drew based on our hypothesis that TRIM40 might be acting as an immunosuppressive protein in bats but not in humans is limited by the lack of a bat TRIM40 in vivo model.

## Figures and Tables

**Table 1:** Primers For Cloning.

**CRISPR**	PV TRIM40-2 F CRISPR	CACCGAGAACAGGGCTTCCGGCAGA
	PV TRIM40-2 R CRISPR	AAACTCTGCCGGAAGCCCTGTTCTC
**Cloning**	hTRIM40 F pCAGGS EcoRI	ATTATAGAATTCATGATCCCTTTGCAGAAGGACAACCA
	hTRIM40 R pCAGGS SmaI	TATAATCCCGGGGAGCTTCTGAGGGGGCTGAAGAAGCA
	PV TRIM40 F EcoRI	ATAATAGAATTCATGTCTCCTTGAGGGAGAACAAC
	PV TRIM40 R SmaI	TATAATCCCGGGCTCAGGTATCAGGTCGTGGCTGTGGGGATGAGCAAAG
	PV TRIM40 C14.17A EcoRI F	ATAATAGAATTCATGGTCTCCTTGAGGGAGAACAACCGGGAAGAGGGCATCGCCCCCATCGCCCAGGAGCAC
	Pt-DDX58-FL-NotI-F	TGATTGCGGCCGCAATGACGGCCGAGGAGCGGCAGAATCTGTAC
	Pt-DDX58-FL-NheI-R	AATCAGCTAGCATTCATCATTTGGGCATTTCTGCAGCATCAAATGGGA
	Pt-IFIH1-FL-NotI-F	TGATTGCGGCCGCAATGTCGAATGAGTATTCTGCAGACAAGAGG
	Pt-IFIH1-FL-NheI-R	AATCAGCTAGCATTCATCAATCTTCATCACTAAACAAACAA

**Table 2:** qPCR Primers.

hEphrin A2 qPCR F	GACCAACGAGACCCTGTACG
hEphrin A2 qPCR R	CTGGGACTAGGAACCCAGGA
hEprhin A5 qPCR F	GGTGTTTCTGGTGCTCTGGA
hEphrin A5 qPCR R	AATCTGGGGTTGCTGCTGTT
hEphrin B1 qPCR F	CTCACACCATCCACCTCCAC
hEphrin B1 qPCR R	AAAAACCCTTCCGACTGCCA
hEphrin B2 qPCR F	TGCTGGGGTGTTTTGATGGT
hEphrin B2 qPCR R	ACCAGTCCTTGTCCAGGTAGA
hEphrin B3 qPCR F	CTCCCTTCCCTTGTGCTCTG
hEphrin B3 qPCR R	GCACATGGGTTCTTGGGGTA
Bat Ephrin A2 F qPCR	AAGTTCCAGCTCTTCACGCC
Bat Ephrin A2 R qPCR	CTGGTGAAGATGGGCTCAGG
Bat Ephrin A5 F qPCR	GTTGCACGTGGAGATGTTGA
Bat Ephrin A5 R qPCR	GTGGTAGTCACCCCTCTGGA
PV Ephrin B1 F qPCR	CCAGAGCAGGAGATTCGCTT
Bat Ephrin B1 R qPCR	TTGCCCGACCTTCATGACAA
PV Ephrin B2 F qPCR	CGCCGGACATTCTGGGAATA
Bat Ephrin B2 R qPCR	TGCTTCCTGTGTCTTCTCCG
Bat Ephrin B3 F qPCR	CTGCCCCAAACCTCCTTCTC
PV Ephrin B3 R qPCR	AGTAATCGTGGTGTGAGCGG
PV TRIM40 F	GTGTCCGGAATCTTTGACAT
PV TRIM40 R	GAGCTGAGTTGCTTTCTCTAA
PV TRIM6 F	TCACCACACGTTCCTCATGG
PV TRIM6 R	CATCTGATTCTTCCAGGATGTTTTC
PA TNFa F	TTCTGCCTGCTGCACTTCGGA
PA TNFa R	TCGGCTTGGGGGTTTGCTACA
PA IL6 F	TCACCTCTCCAAACCAAACC
PA IL6 R	TTTCTGCCATTTTTGGAAGG
EH Mx1 F	GTTACAAAAGTATGGCACAG
EH Mx1 R	AACAGCCGAATGTCGTTGTT
hTRIM40 F	CAACACACTGAAGAATGCTGG
hTRIM40 R	CTTCTGAGGGGGCTGAAGAAG
hTRIM56 F	CTCTGGCTAGTTCTCACAGGTCT
hTRIM56 R	CATGCTGAATGGCCAGGAACT
hTRIM6 F	GGGGTATGCAGCAATTCACT
hTRIM6 R	ACCCAATCACCCAGTATCCA
hTRIM25 F	CGGATGACTGCAAACAGAAA
hTRIM25 R	TCCTTGTCGAGGTGGTCTCT
hMx1 F	GGCTGTTTACCAGACTCCGACA
hMx1 R	CACAAAGCCTGGCAGCTCTCTA
hOAS1 F	GATCTCAGAAATACCCCAGCCA
hOAS1 R	AGCTACCTCGGAAGCACCTT
h18S F	GTAACCCGTTGAACCCCATT
h18S R	CCATCCAATCGGTAGTAGCG
PV TRIM40 F	GTGTCCGGAATCTTTGACAT
EH TRIM40 F	GTGTCCGGAATCCTTGACAT
PV TRIM40 R	GAGCTGAGTTGCTTTCTCTAA
EH TRIM40 R	GAACTGAGTTGCTTTCTCTGA
Pt/EH-TRIM56-RT-F	CCTGGACTGTGCTGATGACTTG
Pt/EH-TRIM56-RT-R	GTCATACCACCCTGCCCTGTA
Pt/EH-TRIM23-RT-F	GGGATGTTGGTGGAAAACACA
Pt/EH-TRIM23-RT-R	GGAGCAAAGCATCTCGGAGT
Pt/EH-TRIM25-RT-F	ACAAACCTTCACGCCCTGTA
Pt-TRIM25-RT-R	GAGTTTGGCTGCAAGGTAGC
EH-TRIM25-RT-R	GAGTTTGGCTGCAAGAGAGT
PV/EH GAPDH-F	ATACTTCTCATGGTTCACAC
PV/EH GAPDH-R	TCATTGACCTCAACTACATG
hGAPDH F	GCAAATTTCCATGGCACCGT
hGAPDH R	GCCCCACTTGATTTTGGAGG

## Data Availability

All data are presented in this manuscript.
